# Preparation of Melamine/Rice Husk Powder Coated Shellac Microcapsules and Effect of Different Rice Husk Powder Content in Wall Material on Properties of Wood Waterborne Primer

**DOI:** 10.3390/polym14010072

**Published:** 2021-12-25

**Authors:** Xiaoxing Yan, Wenbo Li, Yan Han, Taiyu Yin

**Affiliations:** 1 Co-Innovation Center of Efficient Processing and Utilization of Forest Resources, Nanjing Forestry University, Nanjing 210037, China; 2 College of Furnishings and Industrial Design, Nanjing Forestry University, Nanjing 210037, China; liwenbo@njfu.edu.cn (W.L.); hanyan@njfu.edu.cn (Y.H.); yintaiyu@njfu.edu.cn (T.Y.)

**Keywords:** microcapsule, rice husk powder, preparation technology, water-based primer

## Abstract

The melamine/rice husk powder-coated shellac microcapsules were prepared by in-situ polymerization with melamine resin mixed with rice husk powder as microcapsule wall material and shellac as microcapsule core material. The effect of the addition amount of microcapsules with different wall material ratios on the performance of wood waterborne primer coating was investigated. The results show that the most important factor affecting the performance of microcapsules is the content of rice husk powder. Through the preparation and analysis of shellac microcapsule primer coating coated with melamine/rice husk powder, when the content of microcapsule powder is 0–6%, it has little effect on the optical properties of wood waterborne primer coating, and the microcapsule with 5.5% rice husk powder has little effect on the color difference of primer coating. The coating hardness increases with the increase of rice husk powder content in wall material. When the rice husk powder content in wall material is more than 5.5%, the coating hardness reaches the best. When the content of microcapsule powder is 3.0–9.0%, the adhesion of the coating is better, and the coating with rice husk powder content of 5.5% in microcapsule wall material has better impact resistance. When the content of rice husk powder was 5.5% and the content of microcapsule powder was 6%, the elongation at break of the primer coating was the highest and the tensile resistance was the best. The composition of wood waterborne primer did not change after adding microcapsule. The water-based primer with microcapsule has better aging resistance. The water-based primer coating with rice husk powder content of 5.5% and the addition amount of 6% had the best comprehensive performance, which lays the technical reference for the toughness and self-repairing of the waterborne wood coatings.

## 1. Introduction

As a natural resource, wood has the characteristics of lightweight and high strength and is rich in natural color and pattern [1,2,3]. It is widely used in the manufacture of furniture and other daily necessities [4]. Although wood is a renewable resource, its growth cycle is slow, and the wood itself has defects such as wet expansion and dry shrinkage, easy cracking, easy decay, and unstable size [5,6,7]. Therefore, the protection of wood is very important. The traditional method is to paint on the wood surface, which can play a role in protection and beautification [8,9]. Wood waterborne coatings gradually occupy the dominant position in the market because of their safety, non-toxic, wear-resistant, wide applicability, and green environmental protection [10,11]. Wood is a natural polymer heterogeneous composite material with complex composition and structure, it is vulnerable to environmental impact. The toughness of water-based coating is far lower than that of organic solvent coating, especially after drying, the mechanical properties of the coating are greatly reduced, which cannot adapt to the deformation caused by dry shrinkage and wet expansion of wood during use, which leads to microcracks in the coating and reduces the service life of wood products.

Microcapsules are micro containers made of natural or synthetic polymer materials as outer wall materials. The substances loaded inside are called core materials, and the substances wrapped outside are called wall materials [12]. At present, microcapsule self-healing technology is the most widely used method in the research of self-healing intelligent coatings. White et al. [13] proposed the microcapsule self-healing method for the first time. The microcapsule is coated with the repair agent and the catalyst is mixed into the material. When the material is cracked by external stress, the microcapsule mixed in the material breaks, and the released repair agent reacts with the catalyst in the material to repair the crack and realize the self-healing of the material. Sogand et al. [14] prepared microcapsules with ethyl cellulose as shell material and flaxseed oil as core material. The environmentally friendly material ethyl cellulose was successfully applied to the preparation of microcapsules, which improved the environmental pollution caused by traditional chemical synthetic materials as wall materials. Brown et al. [15] used epoxy resin as the base material and added urea-formaldehyde resin to coat DCPD microcapsules. The research shows that when microcapsules are added, the crack can change from flat fracture surface to toothed fracture surface, indicating that the addition of microcapsules can improve the toughness of the base material. These studies show that microcapsules can enhance the mechanical properties of materials and repair the microcracks after the microcracks in coatings. However, there are few studies on the toughness and self-repairing ability of wood furniture and water-based coatings on wood surfaces.

What affects the performance of microcapsules is the choice of capsule wall and core materials. The microcapsule wall material needs to cover the core material and have a certain protective effect on the core material. Secondly, the wall material also needs to be stable in nature, does not release toxic and harmful substances, and does not react with the core material, so as to ensure the stability of the core material for a long time. Melamine resin has a low price and a simple preparation process. It is a thermosetting resin with good mechanical properties. It is often used as the capsule wall material of microcapsules. However, melamine also has the characteristics of insufficient toughness and high brittleness because of its unique triple ring structure [16,17].

Plant fiber is a raw fiber material widely existing in plants. Cellulose is the main component of woody organisms and comes from a wide range of sources. The utilization of cellulose is an important part of the process of sustainable development. At the same time, cellulose has high mechanical properties and has attracted more and more attention from scholars from all walks of life. Vijaykumar Guna et al. [18] strengthened polypropylene composites with rice husk and peanut husk, which significantly improved the tensile strength and flexural strength of the composites. Manshor et al. [19] used durian skin fiber to strengthen PLA biological composite to significantly improve its thermal stability. Rice husk powder, as a by-product of rice, has a wide range of sources, low price, and rich plant fiber.

The selection of core material is the key factor to determine the self-repair ability of microcapsules. Because wood products are not heat-resistant, and the coating microcrack repair process requires room temperature curing. Shellac is a kind of natural resin secreted by a kind of insect called lac insect. Shellac can be cured at room temperature without releasing toxic and harmful gases. Additionally, shellac itself has the characteristics of moisture-proof, rust-proof, anti-corrosion, oil-proof, acid resistance, and strong adhesion. It was also used to repair the surface of wooden furniture [20,21]. Shellac paint can be applied to furniture, floor, doors, and windows, etc. However, there are few studies on pure shellac in microcapsules as a core material [22,23].

Natural cellulose added to the wall material can enhance the toughness of the microcapsule wall material and the water-based coating, inhibit the generation of microcracks, improve the aging resistance effect of the coating, and prolong the effective time of aging resistance and self-repair of the coating. In this paper, the natural cellulose rice husk powder was used to modify melamine wall material, and natural polymer shellac was used as core material to prepare microcapsules to modify wood waterborne coatings. Through the optical test and mechanical test of the waterborne coatings added with microcapsules, the multiple groups of data were recorded and analyzed in combination with the test to study the structure and composition of the wood waterborne paint film. The results provide a technical reference for the application of natural plant fiber in the toughness and self-repair of waterborne wood coatings.

## 2. Materials and Methods

### 2.1. Experimental Materials

The experimental materials were shown in Table 1. Basswood with the specification of 100 mm × 65 mm × 4 mm. After pre-sanding, the surface was smooth, and the color of the wood was uniform. The main components are waterborne acrylic acid copolymer dispersion, matting agent, additive and water, and Dulux waterborne primer with a solids content of about 30.0%.

### 2.2. Preparation and Experimental Design of Microcapsules

#### 2.2.1. Pretreatment of Rice Husk Powder

The 7.5 g rice husk powder was weighed, ground with a mortar, then hydrogen peroxide was added to rice husk powder in the proportion of solid-liquid mass ratio of 1:20. The mixture was mixed evenly, then sodium hydroxide aqueous solution was added to adjust the pH value to about 11.5 and continued stirring in a constant temperature water bath at 70 °C for 24 h to obtain a golden yellow suspension. After cooling to room temperature, the mixture was put into a centrifuge for centrifugation to remove the supernatant. The lower product was put into the drying oven at 60 °C for 4 h.

#### 2.2.2. Core Material Shellac Pretreatment

The 6.64 g shellac was mixed with ethanol in the ratio of 1:5, dissolved, and put into a centrifuge for centrifugation. The upper clear shellac solution was taken for standby.

#### 2.2.3. Preparation of Microcapsules

The mass ratio of core material to wall material is 0.75:1 according to reference [24]. According to the test results, the 5 g melamine raw material and 10 g formaldehyde produced about 8.6 g melamine resin. The L_4_(2^3^) orthogonal test with three factors and two levels was used to prepare melamine/rice husk powder-coated shellac microcapsules. As shown in Table 2, two levels of three factors after treatment were selected: rice husk powder content, water bath temperature, and stirring speed. Among the three factors, the factors that have the greatest impact on the morphology, particle size, yield, and coating rate of microcapsules and the optimal scheme for preparing microcapsules were determined. A single-factor independent experiment was conducted for the most influential factors. The arrangement of orthogonal experimental results is shown in Table 3.

Microcapsule samples of orthogonal experiment No. 1–4 were prepared according to Table 3. Taking sample No. 1 as an example, the preparation process of the microcapsule was described. Firstly, 1 moL of melamine reacts with 3 moL of formaldehyde in an alkaline environment to form a soluble prepolymer, which is further condensed into an insoluble cross-linked product melamine resin. The 5 g of melamine was added into the 10 g of 37% formaldehyde solution and 10 g of deionized water into the beaker according to the mass ratio of 1:2:2, fully stirred in the magnetic stirrer at the rate of 100 r/min, and then the triethanolamine was slowly added to adjust the pH value of the solution to about 9.0. After the melamine is completely dissolved, 0.25 g of rice husk powder and 15 g of deionized water were added and continued to stir for 30 min under a constant temperature water bath at 70 °C. The obtained yellow liquid is melamine/rice husk powder wall material and cooled to 20 °C for use.

The 0.52 g sodium dodecylbenzene sulfonate was added to 51 g water and stirred until it was completely dissolved to obtain an emulsifier with a concentration of 1.0%. The 39.84 g of shellac solution was slowly added to 51.52 g of 1.0% emulsifier at 1200 r/min, stirred, and emulsified at room temperature for 30 min. After the reaction, a stable core solution was obtained.

The wall material of melamine/rice husk powder prepolymer was slowly added to the core material at the rotating speed of 300 r/min, then the citric acid saturated solution was added to adjust the pH value to 2.5–3.0. The reaction is maintained at 20 °C for 3 h, and then placed in a beaker for 7 days. The obtained product is filtered and washed with distilled water to remove the excess emulsifier. Finally, the product is put into a drying oven and dried at 60 °C for 4 h, and melamine/rice husk powder-coated shellac microcapsules were obtained. The detailed preparation process of No. 2–4 microcapsules is the same as that of No. 1, except for the rice husk powder content in wall material.

On the basis of the above orthogonal experiments, the water bath temperature was set at 60 °C, the stirring speed was set as 600 r/min, and the content of rice husk powder in the wall material was variables, which are 0%, 2.8%, 5.5%, 8.0%, and 10.4%, respectively. The dosage of reaction raw materials is shown in Table 4, and the single factor experiment is carried out.

### 2.3. Coating Preparation

Five kinds of microcapsules with different rice husk powder content (0, 2.8%, 5.5%, 8.0%, 10.4%) were added to 4.0 g aqueous primer, respectively. The addition amount of each microcapsule was 0, 3.0%, 6.0%, 9.0%, 12.0% and 15.0%. The Dulux waterborne primer mixed with melamine/rice husk powder-coated shellac microcapsules was stirred evenly for use. The basswood board was polished smoothly, and the primer was evenly coated to the Basswood surface with a film preparer, dried at room temperature for 3 h, polished with 600 mesh fine sandpaper. The above steps were repeated twice, and the coated Basswood was finally placed for 12 h before testing. In the same way, the coating was applied on the glass substrate and peeled from the substrate for a tensile test.

### 2.4. Testing and Characterization

#### 2.4.1. Micro-Morphology Characterization of Microcapsules

The micro-morphology of microcapsules was characterized by Zeiss Axio scope A1 biological microscope. The observation method of the biological microscope is to put a few samples on the slide and cover the slide, and then put them on the observation platform to observe by adjusting the magnification.

#### 2.4.2. Chemical Composition Test of Microcapsules

The chemical composition of microcapsules was analyzed by Fourier transform infrared spectroscopy (FTIR). The test range was 4000–500 cm^−1^, the sample scanning test piece was 16S, and the resolution was 4 cm^−1^. Microcapsule powder and KBr are mixed and crushed for tablet pressing. For coating, the sample is put on the test bench through the ATR method, and the infrared spectrum is obtained through computer analysis connected with the equipment, so as to analyze the composition of the sample.

#### 2.4.3. Test of Coating Rate of Microcapsules

The dried microcapsule was weighed, marked M_1_. After fully grinding the microcapsule powder, the microcapsule powder was soaked in ethanol for 96 h. During this period, the ethanol solvent was replaced every 24 h to fully dissolve the capsule core. The mixture was filtered, weighed after drying, marked *M*_2_. The coating rate *W_J_* was calculated as shown in Formula (1).

(1) $$W_{J}=\frac{M_{1}-M_{2}}{M_{3}}\times 100\%$$

where *M*_1_ − *M*_2_ is the mass of the capsule core contained in the microcapsule, *M*_3_ is the actual amount of microcapsule core material when preparing microcapsules.

#### 2.4.4. Coating Testing and Characterization

The test instruments for this experiment are shown in Table 5. HP-2136 color difference instrument is used to test the color difference of the coating. HG268 intelligent gloss tester was used to test the gloss of the coating. QHQ-A pencil hardness tester is used to measure the hardness of the coating. The coating adhesion was tested by QFH-HG600 film scribing instrument. QCJ-40 coating impact tester is used to test the impact resistance of the coating. The elongation at the break of the coating was measured by a universal mechanical testing machine. The chemical composition of the coating was observed by VERTEX 80 v infrared spectrometer. The surface morphology of the original coating and the aged coating was observed by Quanta-200 environmental scanning electron microscope and Zeiss Axio scope A1 biomicroscope. All experiments were tested four times.

## 3. Experimental Results and Discussion

### 3.1. Morphology Characterization of Microcapsules

**Figure 1:**
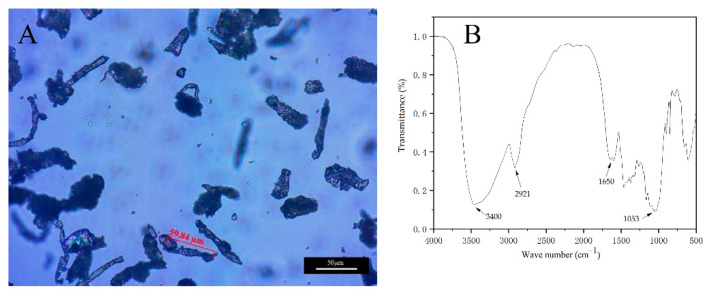
(**A**) Optical micrograph and (**B**) infrared spectrum of treated rice husk powder.

Figure 1 shows the optical micrograph and infrared spectrum of treated rice husk powder. As shown in Figure 1A, the length of treated rice husk powder is about 60 μm. The infrared spectrum detection of the treated rice husk powder is shown in Figure 1B. The wide vibration absorption peak at 3400 cm^−1^ is the stretching vibration peak of O-H. At 2921 cm^−1^, it is the stretching vibration peak of hydrocarbon bond in -CH_3_, 1650 cm^−1^ is the bending vibration peak of water molecule signal -OH in cellulose, and 1053 cm^−1^ is the vibration absorption peak of C-O-C in cellulose. It can be inferred that the main substance in the treated rice husk powder is cellulose.

**Figure 2:**
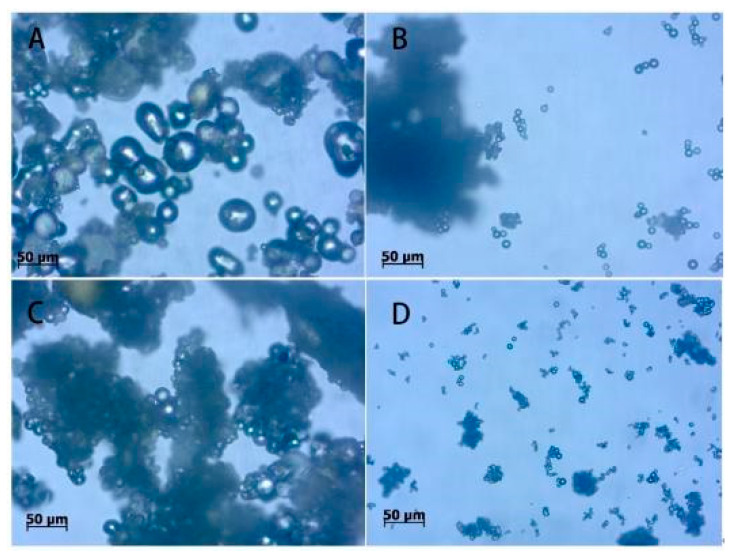
Microscopic morphology of orthogonal test products: (**A**) sample 1, (**B**) sample 2, (**C**) sample 3, (**D**) sample 4.

The morphology of microcapsules orthogonal samples observed by the Zeiss Axio scope A1 biomicroscope is shown in Figure 2. With large particle size, the microcapsules of sample No. 1 (Figure 2A) has irregular particle shape and uneven particle sizes of 30–40 μm or so. Sample 2 (Figure 2B) forms particles with uniform sizes of 6–8 μm or so and high encapsulating efficiency. Sample 3 (Figure 2C) is seriously agglomerated, resulting in different particle sizes of products, with particle sizes of about 15–30 μm. Sample 4 (Figure 2D) is well coated, with less agglomeration and uniform product particles sizes of 5–7 μm. Through the microscopic observation of four samples, it can be seen that both samples No. 2 and No. 4 form microcapsules successfully with high encapsulating efficiency. There are no microcapsules forming in samples No. 1 and No. 3. It is because the low temperature cannot make the prepolymer of melamine and formaldehyde continue to react to form melamine resin as wall material. There is no wall material covering the core material, so the microcapsules cannot be formed [25].

### 3.2. Chemical Composition Analysis of Microcapsules

**Figure 3:**
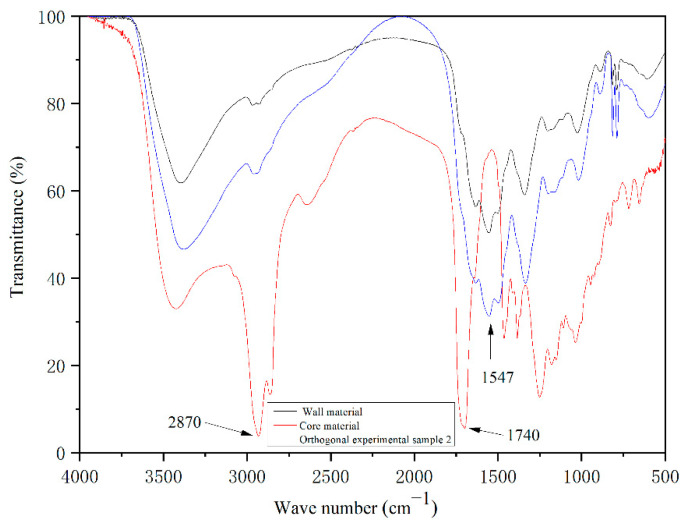
Infrared spectrum of wall material, core material, and microcapsule.

Figure 3 shows the infrared spectra of wall material, core material, and sample No. 2 microcapsule. It can be seen that the core material has a C-H stretching vibration peak at 2870 cm^−1^ and a C=O stretching vibration peak of the carboxyl group at 1740 cm^−1^, which are the characteristic peaks of shellac [26,27]. However, the characteristic peaks of the core material in the infrared spectrum of orthogonal experimental sample No. 2 are weakened to some extent, and the absorption peak at 1547 cm^−1^ belongs to -NH- stretching vibration peak, which is the characteristic peak of melamine resin [28]. It indicates that melamine resin has successfully coated the core material. There is no new characteristic peak in this figure, indicating that the composition of core material and wall material has not been changed. Therefore, melamine/rice husk powder-coated shellac microcapsules were successfully prepared.

### 3.3. Analysis of Microcapsule Yield

The yield and range results obtained by weighing four samples in the orthogonal experiment are shown in Table 6. Mean 1 represents the mean value of the influencing factor at the first level. Mean 2 represents the mean value of the influencing factor at the second level. The range represents the subtraction of the above two mean values for the same influencing factor. Among the 4 samples, sample No. 4 has the maximum weight, which is 6.12 g. The range results showed that the content of rice husk powder had the greatest impact on the microcapsule yield, followed by the water bath temperature. The stirring speed had little effect on the microcapsule yield. The results of morphology analysis showed that microcapsules could not be formed when the water bath temperature was low. Combined with the yield factors, the best preparation process is as follows: water bath temperature of 60 °C, stirring speed of 600 r/min. Then the single factor experiment was designed with the content of rice husk powder as the variable.

### 3.4. Single Factor Experimental Results and Discussion of Microcapsules

**Figure 4:**
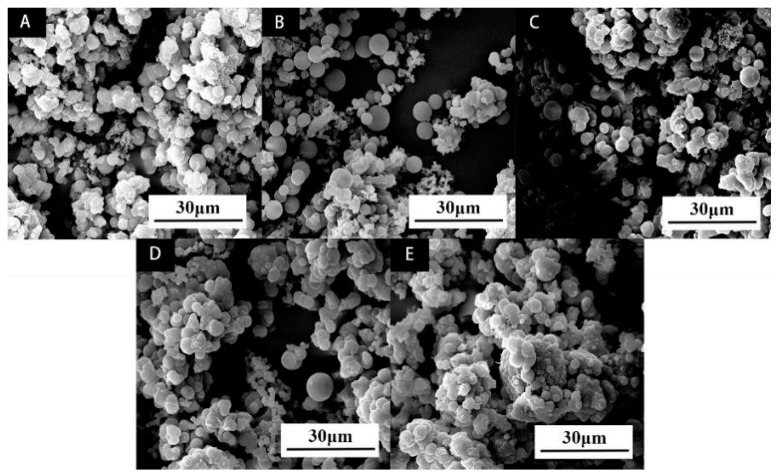
SEM morphology of single factor microcapsules: (**A**) rice husk powder content 0; (**B**) rice husk powder content 2.8%; (**C**) rice husk powder content 5.5%; (**D**) rice husk powder content 8.0%; (**E**) rice husk powder content 10.4%.

The SEM images of microcapsules prepared by single factor optimization experiment of rice husk powder content of wall material are shown in Figure 4 below. Comparing with five microcapsules with different rice husk powder content, the microcapsules without rice husk powder are shown in Figure 4A. It can be observed that there are obvious flocs and less complete spherical substances, which may be due to the poor mechanical properties of the wall material and some damage during mixing. Because the short fiber of cellulose in rice husk powder will increase the surface roughness of microcapsules, it can be seen that with the increase of rice husk powder content, some flocs will appear on the surface of microcapsules. The microcapsules with rice husk powder content of 5.5% are round and have an average particle size of about 6 μm. It was observed that the microcapsules containing 2.8%, 5.5%, and 8.0% of rice husk powder content in the wall materials (Figure 4B–D) are relatively less agglomerated by SEM.

**Figure 5:**
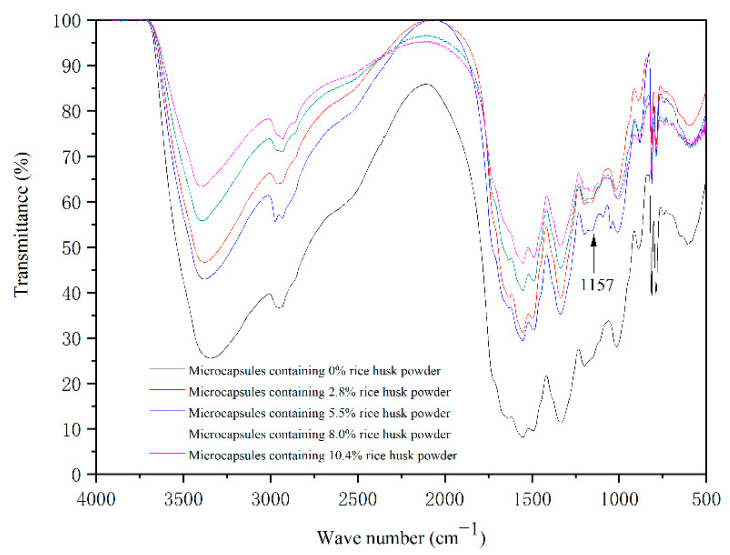
Infrared spectrum of microcapsules in the single-factor test.

Figure 5 shows the infrared spectra of five microcapsules prepared by a single factor independent experiment. The absorption peak at 1547 cm^−1^ belongs to -NH- stretching vibration peak, which belongs to the characteristic peak of melamine resin. The absorption peak at 1157 cm^−1^ in the infrared spectrum of microcapsules with rice husk powder in the wall material is split and the peak shape is changed. It can be inferred that this peak is affected by the C-H vibration of the aromatic core and the C-O-C antisymmetric “bridge” stretching vibration peak in rice husk powder. It can be judged that the wall material of the microcapsule contains cellulose [29]. The absorption peaks at other positions of the infrared spectra of all microcapsules are consistent with those of the microcapsules with a content of 2.8% rice husk powder. It can be concluded that the microcapsules are successfully prepared, and the chemical composition of the microcapsules has not changed.

The microcapsule quality obtained by unit mass core material is taken as the evaluation result, as shown in Table 7. The low quality of the product may be due to the low conversion of core and wall materials. It can be seen from Table 7 that the microcapsule yield first increased and then decreased with the increase of rice husk powder content. The reason is that the rice husk powder increases the toughness of the wall material, and the wall material is not easy to crack during the preparation of microcapsules, so the output of microcapsules formed by coating increases. However, if the content of rice husk powder is too high, it will agglomerate during the formation of microcapsules, making the capsule wall materials adhere to each other. The core materials cannot be evenly dispersed during the mixing process, so as to reduce the output of microcapsules [30].

The coating rate results of microcapsules with different rice husk powder content are shown in Table 8. With the increase of rice husk powder content in wall material, the microcapsule coating rate increased first and then decreased. When the content of rice husk powder in the wall material increased from 0 to 8.0%, the coating rate increased from 20% to 47%, increasing by 27%. When the content of rice husk powder in the microcapsule wall material reached 10.4%, compared with 8.0%, the coating rate decreased from 47% to 31%, decreased by 16.0%. Because the cellulose of rice husk powder in the microcapsule wall material can enhance the toughness of the wall material, making the microcapsules difficult to crack during the synthesis and stirring process, and the high content of rice husk powder leads to the reduction of melamine resin content in the wall material. The wall material of microcapsule agglomerates seriously, which cannot completely wrap the core material, resulting in the decrease of coating rate [31].

### 3.5. Effect of Different Rice Husk Powder Content in Wall Material on Properties of Wood Waterborne Primer

The color difference value of the coating is the main basis for judging whether the coating color is uniform. “*L*” represents brightness, “*a*” represents the red-green difference, “*b*” represents the yellow-blue difference. The date groups of *L*_1_, *a*_1_, and *b*_1_ are obtained at one point on the coating by a color difference meter. Another group of data *L*_2_, *a*_2,_ and *b*_2_ are obtained at another point on the coating. The color difference is calculated according to Formula (2).

(2) $$\Delta E=\sqrt{{\left(\Delta L\right)}^{2}+{\left(\Delta a\right)}^{2}+{\left(\Delta b\right)}^{2}}$$

where: $\Delta L=L_{1}-L_{2}$, $\Delta a=a_{1}-a_{2}$, $\Delta b=b_{1}-b_{2}$.

**Figure 6:**
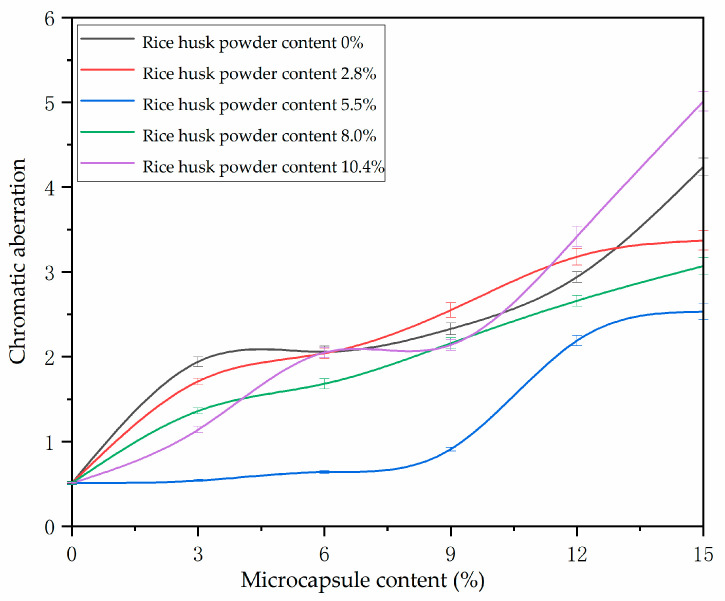
Effect of microcapsules with different contents of rice husk powder on the color difference.

According to Figure 6, under the horizontal comparison of microcapsules with the same content of rice husk powder, the color difference value of the coating improves with the increase of the content of microcapsules in the coating. This is because the prepared microcapsules are milky yellow powder. When the addition amount is high, it will change the transparency of the coating and the color of the primer coating to affect the color difference value. The influence of microcapsules with different rice husk powder content on the color difference is not obvious. The color difference value of microcapsules with 2.8% rice husk powder in wall material increased from 0.51 to 3.37, and the color difference value of microcapsules with 5.5% rice husk powder in wall material increased from 0.51 to 2.53. The reason is that the particle size of microcapsules is small and uniform. It is easy to be evenly distributed in the coating, so the influence on the color difference is low. With the content of microcapsules between 0~9%, the content of microcapsules has little effect on the color difference of waterborne primer coating. In this range, the color difference of microcapsules with rice husk powder content of 5.5% increased from 0.51 to 0.64, which had the least effect on the color difference of primer coating.

**Figure 7:**
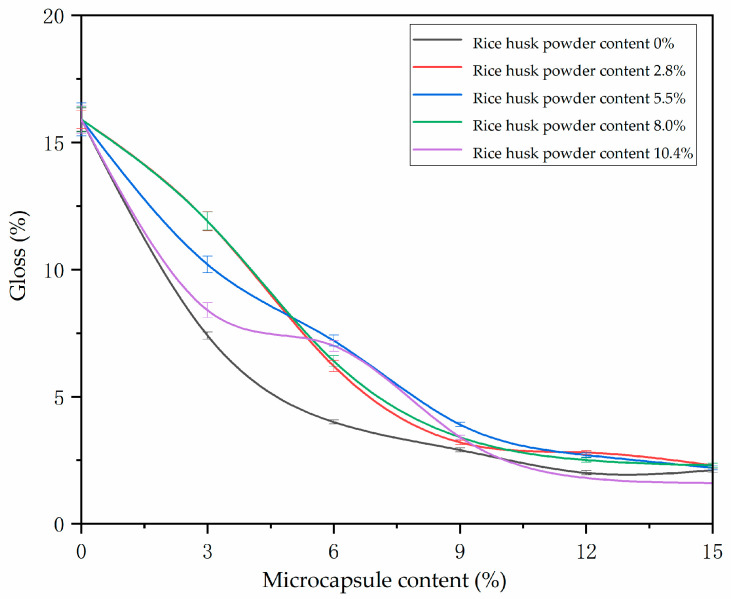
Effect of microcapsules with different wall material ratios on the gloss of waterborne primer coating.

Gloss is to test the specular reflection ability of the coating surface to light, and it is the judgment basis of the smoothness of the coating surface. The test results are shown in Figure 7. It can be observed from Figure 7 that the gloss of the coating decreases with the increase of microcapsule content. This is because the microcapsules are spherical solid powders. The increase of microcapsule content in the coating will increase the roughness of the coating surface, resulting in diffuse reflection of light and low gloss of the coating surface. When the microcapsule content is 0~6%, it has the least effect on coating gloss.

The mechanical properties of waterborne coatings on wood surfaces are generally low. The prepared microcapsules with different content of rice husk powder in wall materials were added into the water-based primer for the hardness test, and the results are shown in Table 9. The hardness of the coating increases with the increase of microcapsule content. Under the condition of different rice husk powder content, when the rice husk powder content in wall material is more than 5.5%, the coating hardness reached 6H, indicating that the coating hardness increases with the increase of rice husk powder content in wall material.

The adhesion of water-based primer coatings with different microcapsules and different contents was tested by a film scribing instrument. The results are shown in Table 10. When the amount of microcapsule with rice husk powder in wall materials is 3–9%, the adhesion level of the coating is level 1 and level 2. That is, when the amount of microcapsules is 3–9%, the adhesion of the coating is better. When the content of rice husk powder remains unchanged, the adhesion of primer coating gradually decreases with the increase of microcapsule addition, which is due to the increase of microcapsule addition, resulting in more particles in the coating, which make the decrease of interfacial adhesion between coating and wood and between coating and coating, so as to reduce the adhesion of coating [32].

The impact resistance of the prepared waterborne primer coating is tested by the coating impactor tester, and the test results are shown in Table 11. The impact resistance of the coating first increases and then decreases with the increase of microcapsule content. When the content of microcapsules is 6–12%, the impact strength of the coating containing 5.5% and 8.0% rice husk powder in the microcapsule wall material is above 10 kg·cm. It may be because the microcapsule increases the mechanical properties of the coating. However, with the increase of microcapsule concentration, the microcapsule will agglomerate in the coating, which reduces the adhesion of the coating and is difficult to withstand the impact of external forces. Thus, the impact resistance of the coating is reduced [33].

**Figure 8:**
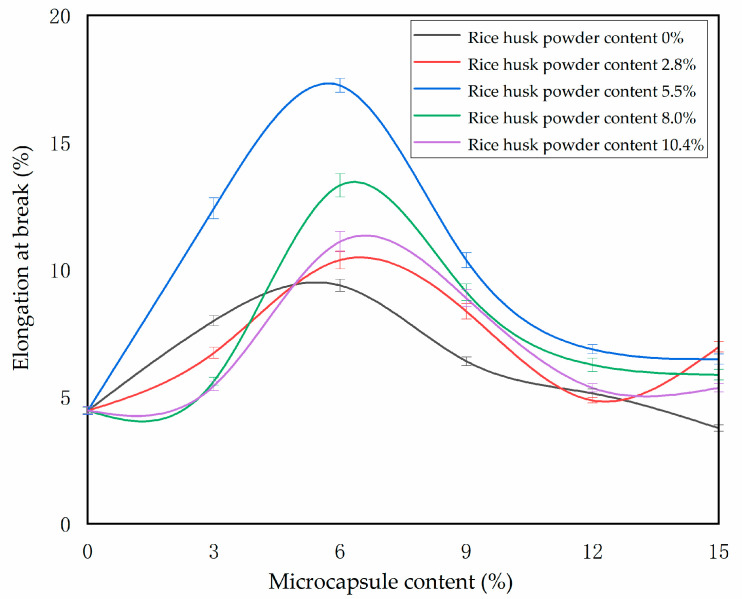
Effect of microcapsules with different contents of rice husk powder on elongation at break of waterborne primer coating.

The elongation at the break of the waterborne primer is measured by the universal mechanical testing machine. The calculation results are shown in Figure 8. It can be seen that the elongation at the break of the primer coating increases first and then decreases with the increase of the content of microcapsules in the coating. The wall material of the microcapsule contains rice husk powder, which is plant cellulose. Adding it to the waterborne primer will significantly improve the toughness of the coating itself. Once the content of rice husk powder increases, the particle size of microcapsules is uneven and easy to agglomerate, which will make the coating uneven and brittle to reduce the toughness and easy to fracture [34]. When the amount of rice husk powder was 5.5% and the amount of microcapsule was 6.0%, the elongation at break of the primer coating is the highest 17.24%, which increases by 12.79%. and the tensile property is the best.

**Figure 9:**
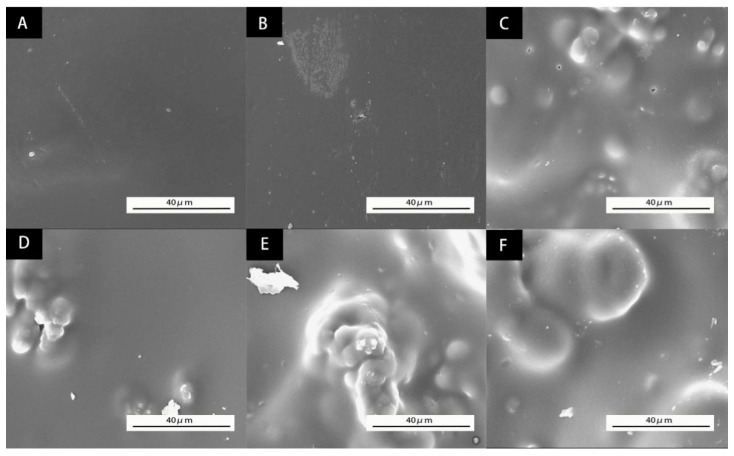
SEM of waterborne coatings with different contents of microcapsules (rice husk powder content 5.5%): (**A**) 0%; (**B**) 3%; (**C**) 6%; (**D**) 9%; (**E**) 12%; (**F**) 15%.

Based on the above test results, the primer coating with the microcapsules containing 5.5% rice husk powder in the wall material has excellent optical and mechanical properties. The microstructure of primer coatings with different contents of microcapsules (rice husk powder content 5.5%) was observed by environmental scanning electron microscope, as shown in Figure 9. It can be observed that the surface of the primer coating without microcapsules is smooth. With the increase of microcapsule content, the surface of the coating became rough. When the microcapsule content is less than 6%, the microcapsule particles are evenly dispersed in the coating without obvious protrusions. For the coating with microcapsule content greater than 6%, the microcapsules can be observed to form agglomeration and many protrusions on the coating surface.

**Figure 10:**
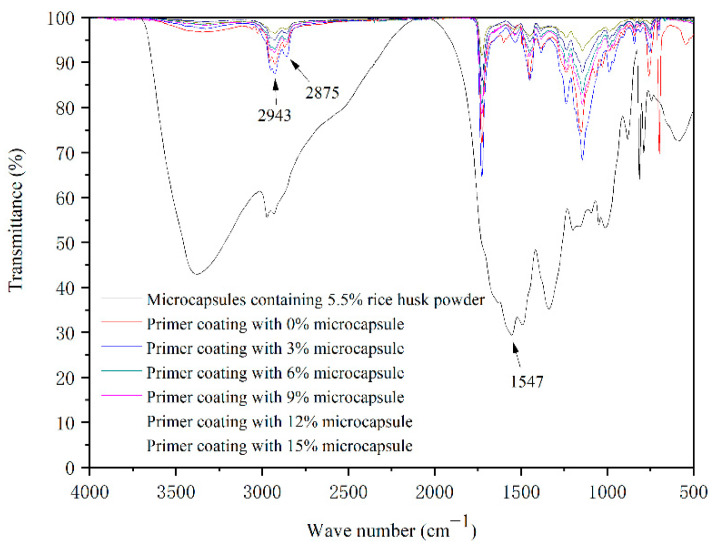
Infrared spectrum of waterborne primer with different contents of microcapsules.

The microcapsules with rice husk powder content of 5.5% and the waterborne primer coatings with different contents of microcapsules (rice husk powder content of 5.5%) were detected by Fourier transform infrared spectrometer. The results are shown in Figure 10. At 2943 cm^−1^ and 2875 cm^−1^, they are the characteristic absorption peaks of methyl and methylene, which are mainly the characteristic chemical bonds of waterborne acrylic acid (the main component of the primer) [35]. The absorption peak at 1547 cm^−1^ belongs to -NH- stretching vibration peak, which belongs to the characteristic peak of melamine resin [36]. There is an absorption peak in the aqueous coating with microcapsules, which proves that microcapsules exist in the coating. Other characteristic absorption peaks of the coating do not disappear, indicating that the microcapsules do not produce a chemical reaction with the coating, and the coating composition is still stable after adding microcapsules.

**Figure 11:**
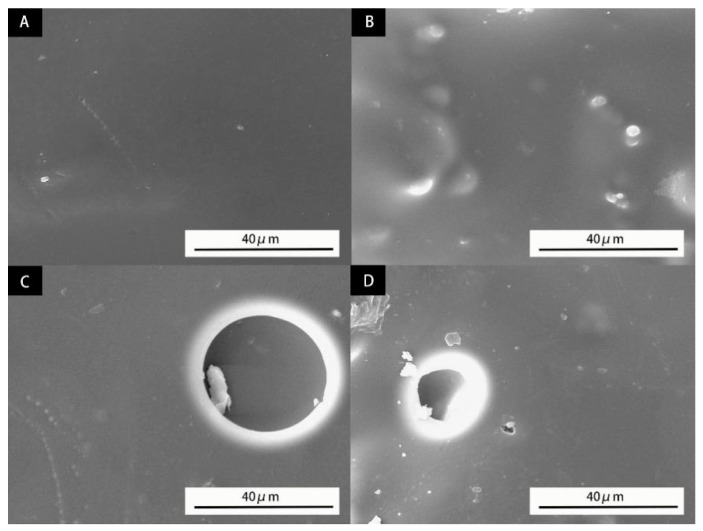
SEM of waterborne primer coating with different content of microcapsules before and after aging; before aging: (**A**) 0% of the microcapsule, (**B**) 6% of microcapsule; after aging: (**C**) 0% of the microcapsule, (**D**) 6% of the microcapsule.

The waterborne primer with 6.0% microcapsules addition has good toughness, optical properties, and mechanical properties. Therefore, this sample is selected for the aging resistance test and compared with the blank sample. After UV aging, the color difference of the primer without microcapsules changes more, and the gloss decreases more obviously than that of the primer with 6% microcapsules, indicating that adding microcapsules to the waterborne primer coating can improve the aging resistance of the coating (Table 12). In Figure 11, it can be observed that almost no particles can be seen in the primer coating without microcapsules before aging. After aging, it can be clearly observed that the primer coating without microcapsules has large bubbles with a diameter of 40 μm. The bubble diameter of primer coating with 6% microcapsule is only 15 μm, which further proves that microcapsules increase the stability and aging resistance of waterborne primer coating. This is because the surface of the coating will be damaged by bubbles during aging. According to the previous measurement of the elongation at the break of the coating, the coating with microcapsules has strong toughness, so it is not easy to crack after bubbles are generated. After cracking, the outflow of cystic substances at the crack will reduce the crack to stabilize the surface optical resistance of the coating and reduce the bubble size.

**Figure 12:**
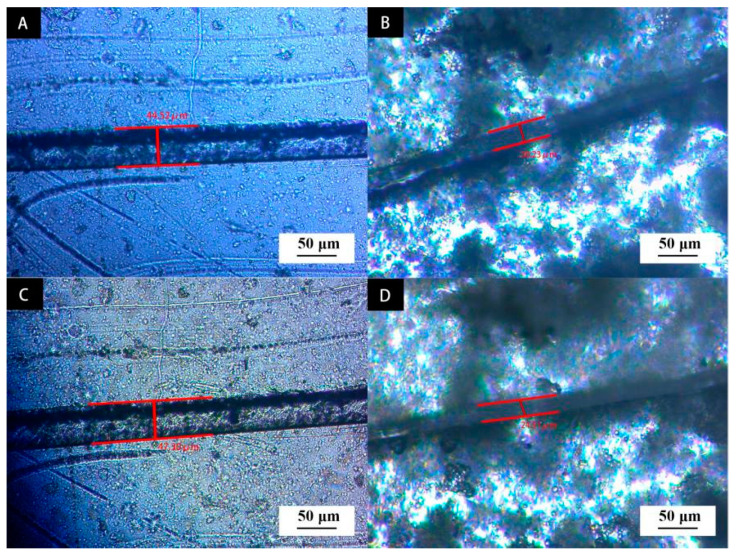
Scratch test of coating: (**A**) microcapsule content 0, scratch first day, (**B**) microcapsule content 6.0%, scratch first day; (**C**) microcapsule content 0, scratch fifth day, (**D**) microcapsule content 6.0%, scratch fifth day.

The scratch control experiment was carried out between the waterborne primer coating with 6.0% microcapsule and without microcapsule. The scratches on the first and fifth days were recorded respectively. The results are shown in Figure 12. Figure 12A,C show the coating scratches without microcapsules. It can be seen that the scratches increased by 2.78 μm after 5 d. This is because the crack will gradually expand under the influence of environmental factors. Figure 12B,D show the primer coating added with 6.0% microcapsules. The scratch was reduced from 30.23 μm to 24.51 μm after 5 d with scratch size reduction of 5.72 μm. The repair rate was about 18.9%. When the external load leads to the cracking of the waterborne primer, the microcapsule wall at the crack is torn, and the repair components of the capsule core flow from the microcapsule to the crack under the siphon action. The repair agent core material shellac is naturally physically cured at room temperature, which can block and bridge the microcracks to reduce the cracks of the waterborne coating to a certain extent.

## 4. Conclusions

The best preparation process for melamine/rice husk powder-coated shellac microcapsules was as follows: water bath temperature of 60 °C, stirring speed of 600 r/min, the content of rice husk powder in the wall materials of 5.5–8.0%. When the microcapsules containing 5.5% rice husk powder in the wall material were added to the water-based primer at the content of 6.0%, the comprehensive performance of the water-based primer coating was the best with the color difference of 0.13, the gloss changed by 8.7% at 60° incidence angle, the hardness of 6H, the adhesion of level 1, the impact resistance of 15 kg·cm, and the elongation at break of 17.24%. The aging resistance test shows that the color difference and gloss of the coating change little. When the primer cracks, it has a self-repairing effect, and the repair rate is about 18.9%. The addition of rice husk powder increases the toughness of the primer coating, which can inhibit the cracking of the primer coating to achieve better protection for wood products and reduce unnecessary economic losses. It can be provided that a technical reference for the application of self-repairing and toughening dual-functional wood product surface coatings.

## Figures and Tables

**Table 1:** List of experimental materials.

Experimental Materials	Molecular Mass (g/mol)	CAS	Manufacturer
5% hydrogen peroxide	34.01	7722-84-1	Nanjing Chemical Reagent Co., Ltd., Nanjing, China
sodium hydroxide	40.00	1310-73-2	Nanjing Chemical Reagent Co., Ltd., Nanjing, China
99.9% melamine	126.12	108-78-1	Shuntian Chemical Co., Ltd., Shandong, China
37% formaldehyde	30.03	50-00-0	Nanjing Chemical Reagent Co., Ltd., Nanjing, China
triethanolamine	149.1882	102-71-6	Nanjing Chemical Reagent Co., Ltd., Nanjing, China
sodium dodecyl benzene sulfonate	348.476	25155-30-0	Tianjin Beichen Fangzheng Reagent Factory, Tianjin, China
citric acid monohydrate	210.14	5949-29-1	Tianjin Beilian Fine Chemicals Development Co., Ltd., Tianjin, China
absolute ethanol	46.07	64-17-5	Hangzhou Outop Biotechnology Co., Ltd., Hangzhou, China
100 mesh rice husk powder	-	-	Lianfeng Agricultural Products Co., Ltd., Lianyungang, China
shellac	-	-	Jinan Dahui Chemical Technology Co., Ltd., Jinan, China
Dulux waterborne primer	-	-	Shanghai Keyuan Industrial Co., Ltd., Shanghai, China

**Table 2:** Influencing factors and levels.

Level	Rice Husk Powder Content (%)	Water Bath Temperature (°C)	Stirring Speed (r)
1	2.8	20	300
2	8.0	60	600

**Table 3:** Orthogonal test arrangement.

Sample	Rice Husk Powder Content (%)	Water Bath Temperature (°C)	Stirring Speed (r)
1	2.8	20	300
2	2.8	60	600
3	8.0	20	600
4	8.0	60	300

**Table 4:** List of consumption of each substance in the single-factor test.

Rice Husk Powder Content (%)	Rice Husk Powder (g)	Melamine (g)	37% Formaldehyde Solution (g)	Shellac (g)
0	0	5.00	10.00	6.45
2.8	0.25	5.00	10.00	6.64
5.5	0.50	5.00	10.00	6.83
8.0	0.75	5.00	10.00	7.01
10.4	1.00	5.00	10.00	7.20

**Table 5:** Experimental equipment list.

Experimental Equipment	Manufacturer
HP-2136 color difference instrument	Hangzhou Caipu Technology Co., Ltd., Hangzhou, China
HG268 intelligent gloss tester	Shenzhen Sanenshi Technology Co., Ltd., Shenzhen, China
QHQ-A Pencil hardness tester	Dongguan Dalai Instrument Co., Ltd., Dongguan, China
QFH-HG600 film scribing instrument	Dongguan Huaguo Precision Instrument Co., Ltd., Dongguan, China
QCJ-40 coating impact tester	Dongguan Weida Instrument Co., Ltd., Dongguan, China
Universal mechanical testing machine	Dongguan Hongjin Testing Instrument Co., Ltd., Dongguan, China
Quanta-200 scanning electron microscope	Shenzhen Chenqixi Trading Co., Ltd., Shenzhen, China
Zeiss Axio scope A1 biomicroscope	Shenzhen Chenqixi Trading Co., Ltd., Shenzhen, China
VERTEX 80 V infrared spectrometer	Guangzhou Huruiming Instrument Co., Ltd., Guangzhou, China

**Table 6:** Results of output of microcapsules.

Sample	Rice Husk Powder Content (%)	Water Bath Temperature (°C)	Stirring Speed (r)	Yield (g)
1	2.8	20	300	2.64
2	2.8	60	600	3.87
3	8.0	20	600	5.21
4	8.0	60	300	6.12
Mean 1	3.225	3.925	4.38	
Mean 2	5.665	4.995	4.54	
Range	2.44	1.07	0.16	

**Table 7:** Yield of microcapsules in the single-factor test.

Sample	Rice Husk Powder Content (%)	Mass of Core Material (g)	Yield (g)
1	0	6.45	0.55
2	2.8	6.64	0.58
3	5.5	6.83	1.08
4	8.0	7.01	1.06
5	10.4	7.20	1.04

**Table 8:** Results of microcapsule coating rate in the single-factor test.

Sample	Rice Husk Powder Content (%)	Mass of Core Material (g)	Coating Rate (%)
1	0	6.45	20
2	2.8	6.64	23
3	5.5	6.83	35
4	8.0	7.01	47
5	10.4	7.20	31

**Table 9:** Effect of microcapsule addition on the hardness of waterborne primer coating.

Microcapsule Addition (%)	Hardness				
	Rice Husk Powder Content of 0	Rice Husk Powder Content of 2.8%	Rice Husk Powder Content of 5.5%	Rice Husk Powder Content of 8.0%	Rice Husk Powder Content of 10.4%
0	H	H	H	H	H
3	3H	4H	5H	5H	6H
6	4H	5H	6H	6H	6H
9	4H	6H	6H	6H	6H
12	5H	6H	6H	6H	6H
15	5H	6H	6H	6H	6H

**Table 10:** Effect of microcapsule addition on adhesion of waterborne primer coating.

Microcapsule Addition (%)	Adhesion (Level)				
	Rice Husk Powder Content of 0	Rice Husk Powder Content of 2.8%	Rice Husk Powder Content of 5.5%	Rice Husk Powder Content of 8.0%	Rice Husk Powder Content of 10.4%
0	0	0	0	0	0
3	2	1	1	1	1
6	3	1	1	2	2
9	3	2	1	2	2
12	3	3	2	3	3
15	3	3	2	3	3

**Table 11:** Effect of microcapsule content on the impact resistance of waterborne primer coating.

Microcapsule Addition (%)	Impact Strength (kg·cm)				
	Rice Husk Powder Content of 0	Rice Husk Powder Content of 2.8%	Rice Husk Powder Content of 5.5%	Rice Husk Powder Content of 8.0%	Rice Husk Powder Content of 10.4%
0	5	5	5	5	5
3	5	5	7	6	6
6	10	10	15	13	10
9	10	10	10	10	10
12	10	10	10	10	10
15	5	7	7	7	7

**Table 12:** Changes of color difference and glossiness of water-based primer with different contents of microcapsules after the aging test.

Microcapsule Addition (%)	State	L	a	b	Chromatic Aberration	Gloss (%)
0	Before aging	66.0	18.3	29.6	-	15.9
	After aging	66.7	17.9	27.5	4.34	13.9
6	Before aging	70.9	14.2	29.5	-	7.2
	After aging	71.5	15.5	32.3	1.59	6.5

## Data Availability

Not applicable.
